# Identification and validation of aging-related genes in COPD based on bioinformatics analysis

**DOI:** 10.18632/aging.204064

**Published:** 2022-05-24

**Authors:** Shan Zhong, Li Yang, Naijia Liu, Guangkeng Zhou, Zhangli Hu, Chengshui Chen, Yun Wang

**Affiliations:** 1 Guangdong Key Laboratory of Epigenetics, College of Life Sciences and Oceanography, Shenzhen University, Shenzhen 518055, P.R. China; 2 Key Laboratory of Optoelectronic Devices and Systems of Ministry of Education and Guangdong Province, College of Optoelectronic Engineering, Shenzhen University, Shenzhen 518061, P.R. China; 3 Key Laboratory of Interventional Pulmonology of Zhejiang Province, The First Affiliated Hospital of Wenzhou Medical University, Wenzhou 325015, P.R. China; 4 Longhua Innovation Institute for Biotechnology, College of Life Sciences and Oceanography, Shenzhen University, Shenzhen 518060, P.R. China

**Keywords:** COPD, aging, biomarker, bioinformatics analysis

## Abstract

Chronic obstructive pulmonary disease (COPD) is a serious chronic respiratory disorder. One of the major risk factors for COPD progression is aging. Therefore, we investigated aging-related genes in COPD using bioinformatic analyses. Firstly, the Aging Atlas database containing 500 aging-related genes and the Gene Expression Omnibus database (GSE38974) were utilized to screen candidates. A total of 24 candidate genes were identified related to both COPD and aging. Using gene ontology and Kyoto Encyclopedia of Genes and Genomes enrichment analyses, we found that this list of 24 genes was enriched in genes associated with cytokine activity, cell apoptosis, NF-κB and IL-17 signaling. Four of these genes (*CDKN1A, HIF1A, MXD1* and *SOD2*) were determined to be significantly upregulated in clinical COPD samples and in cigarette smoke extract-exposed Beas-2B cells *in vitro*, and their expression was negatively correlated with predicted forced expiratory volume and forced vital capacity. In addition, the combination of expression levels of these four genes had a good discriminative ability for COPD patients (AUC = 0.794, 95% CI 0.743–0.845). All four were identified as target genes of hsa-miR-519d-3p, which was significantly down-regulated in COPD patients. The results from this study proposed that regulatory network of hsa-miR-519d-3p/*CDKN1A, HIF1A, MXD1,* and *SOD2* closely associated with the progression of COPD, which provides a theoretical basis to link aging effectors with COPD progression, and may suggest new diagnostic and therapeutic targets of this disease.

## INTRODUCTION

Chronic obstructive pulmonary disease (COPD) is a complicated and heterogeneous respiratory condition with a high morbidity and mortality over three million people died from this disease worldwide per year, which causes a huge burden to medical and financial systems globally [1, 2]. Although previous studies reported that the main risks for COPD are long-term exposure to cigarette smoke or air pollution and genetic factors, the aging process is also important in the pathogenesis of COPD [3–6]. A recent study indicated that the prevalence of COPD increases with age (5.1% in 35–54 year-olds, 13.3% in 55–64 year-olds, and 21.7% in those older than 65) [7] and patients older than 65 year-old had a five-fold increase at risk of COPD process compared to patients younger than 40 [8]. Investigation of the relationship between aging associated genes and progression of COPD may provide new biomarkers for diagnosis and personalized therapy of this disease.

Aging is the important physiological and pathophysiological processes in human life, which involves inflammation, oxidative stress, mitochondrial dysfunction, epigenetic alterations, cell senescence and death, and regulates COPD development. Dysregulation of aging-related genes, such as *SIRT1* and *SIRT6*, were recently demonstrated to be associated with COPD [9–14]. Previous studies showed that the reduction of *SIRT1* and *SIRT6* expression could exacerbate the response to oxidative stress, premature senescence and chronic inflammation, which further accelerate the aging process in the lung [11–13]. The activity of *SIRT1* may be partially enhanced by treatment with the anti-inflammatory molecule Resveratrol to reverse the progression of CSE-induced COPD [14]. Li et al. found that Klotho, an anti-aging protein, could inhibit the expression of inflammatory mediators such as MMP-9, TNF-α, and IL-6 via the NF-κB pathway in COPD [15]. Another study demonstrated that Klotho down-regulation in COPD was associated with accelerated lung aging in COPD development and increased oxidative stress, inflammation, and apoptosis of airway epithelial cells [16]. However, the roles of aging-related genes during COPD development remain largely unknown.

MicroRNAs (miRNAs) are small non-coding RNA 18–22 nucleotides in length that block protein translation through miRNA-mRNA interactions, or increase mRNA degradation [17]. Abnormal miRNA expression is important in cancer and other human diseases due to their importance in pathophysiological processes and several miRNA-based therapeutics have applied to clinical testing, such as miR-34 mimic reached phase I clinical trials for cancer treatment and miR-122 antagonist reached phase II trials for hepatitis treatment [18, 19]. The roles of miRNA in COPD have been examined over the past few years [20, 21]. Several studies highlighted that miR-34a was up-regulated in patients with severe COPD and involved in the pathogenesis of COPD by affecting the HIF-1α-dependent lung structure maintenance program [22], regulating the apoptosis of human pulmonary microvascular endothelial cells by directly targeting *Notch1* [23], and orchestrating the oxidative stress response by regulating the expression of *SIRT1* and *SIRT6* [24]. Another study indicated that elevated miR-125a-5p facilitated the senescence of lung epithelial cells to participate in the pathogenesis of smoking-induced COPD [25].

**Figure 1:**
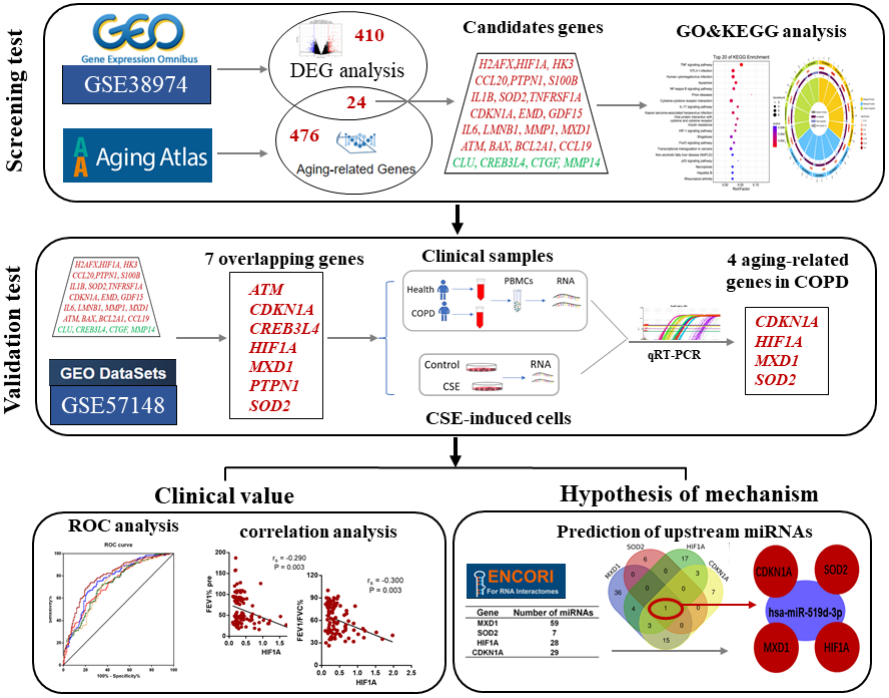
The work-flow of this study.

The development and application of high-throughput sequencing technology and multiple public databases have facilitated for screening new disease-related biomarkers using enrichment analyses of gene expression profiles from the gene expression omnibus (GEO) database [26]. In this study, we used gene expression profiles of COPD, aging related databases, bioinformatic analyses and validation tests to screen aging-related genes as biomarkers of COPD development, and to investigate the effects of miRNAs on these candidate aging-related genes. A workflow of this study is shown in Figure 1.

## RESULTS

### Screening candidate aging-related genes in COPD

**Figure 2:**
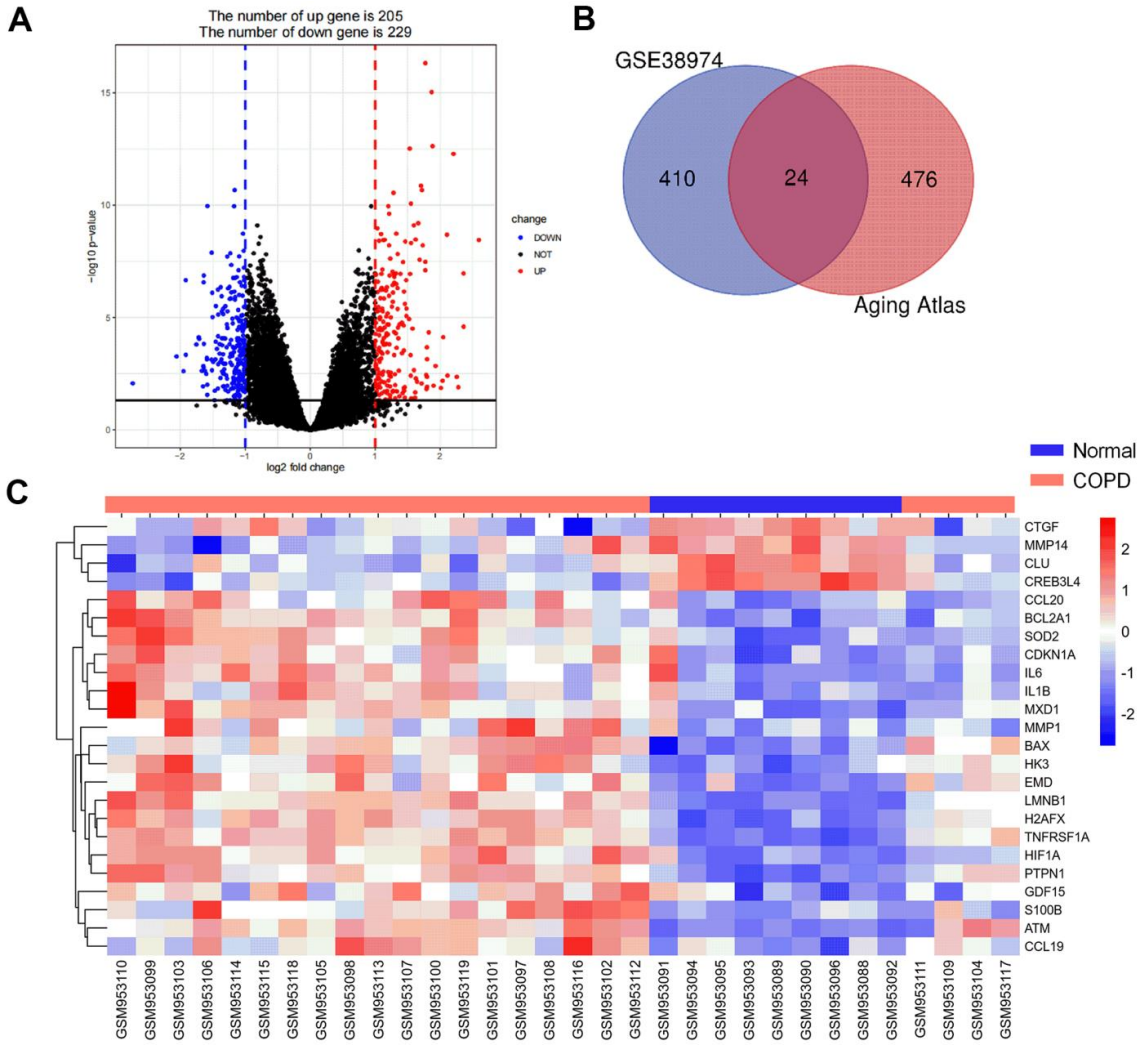
**Aging-related differentially expressed genes (DEG) between COPD subjects and normal controls.** (**A**) Volcano plot of the DEGs from GSE38974 with adjusted *P* <0.05 and |log_2_ FC| >1 as threshold values. Red and blue dots indicate significantly upregulated and downregulated genes, respectively. (**B**) Venn diagram of COPD differentially expressed genes and aging-related genes. (**C**) Heatmap of the expression of 24 aging- and COPD-related genes.

**Figure 3:**
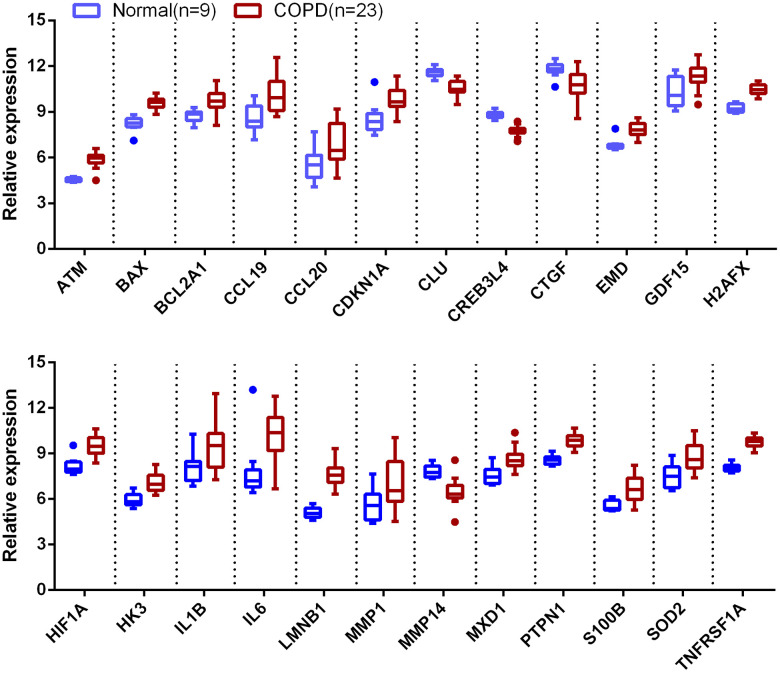
**Boxplot of 24 aging-related genes in COPD and normal subjects.** Data obtained from GSE38974 and presented as probe intensity.

The Aging Atlas database and GSE38974 data set which contained lung tissues of 9 normal and 23 COPD patients were used to screen candidate genes. Firstly, a total of 434 differentially expressed genes (DEGs; 205 upregulated and 229 downregulated) were identified between COPD and normal samples in the GSE38974 dataset, using |log_2_FC| > 1 and adjusted *P* < 0.05 as cutoff values (Figure 2A). Secondly, these 434 DEGs were compared with 500 aging-related genes from the Aging Atlas database, and identified 24 genes which were present in both (Figure 2B, 2C). Among these 24 genes, 20 were upregulated (*ATM*, *BAX*, *BCL2A1*, *CCL19*, *CCL20*, *CDKN1A*, *EMD*, *GDF15*, *H2AFX*, *HIF1A*, *HK3*, *IL1B*, *IL6*, *LMNB1*, *MMP1*, *MXD1*, *PTPN1*, *S100B*, *SOD2*, and *TNFRSF1A*), and 4 were downregulated (*CLU*, *CREB3L4*, *CTGF*, and *MMP14*; Figure 3).

### Functional annotation of candidate aging-related genes in COPD

**Figure 4:**
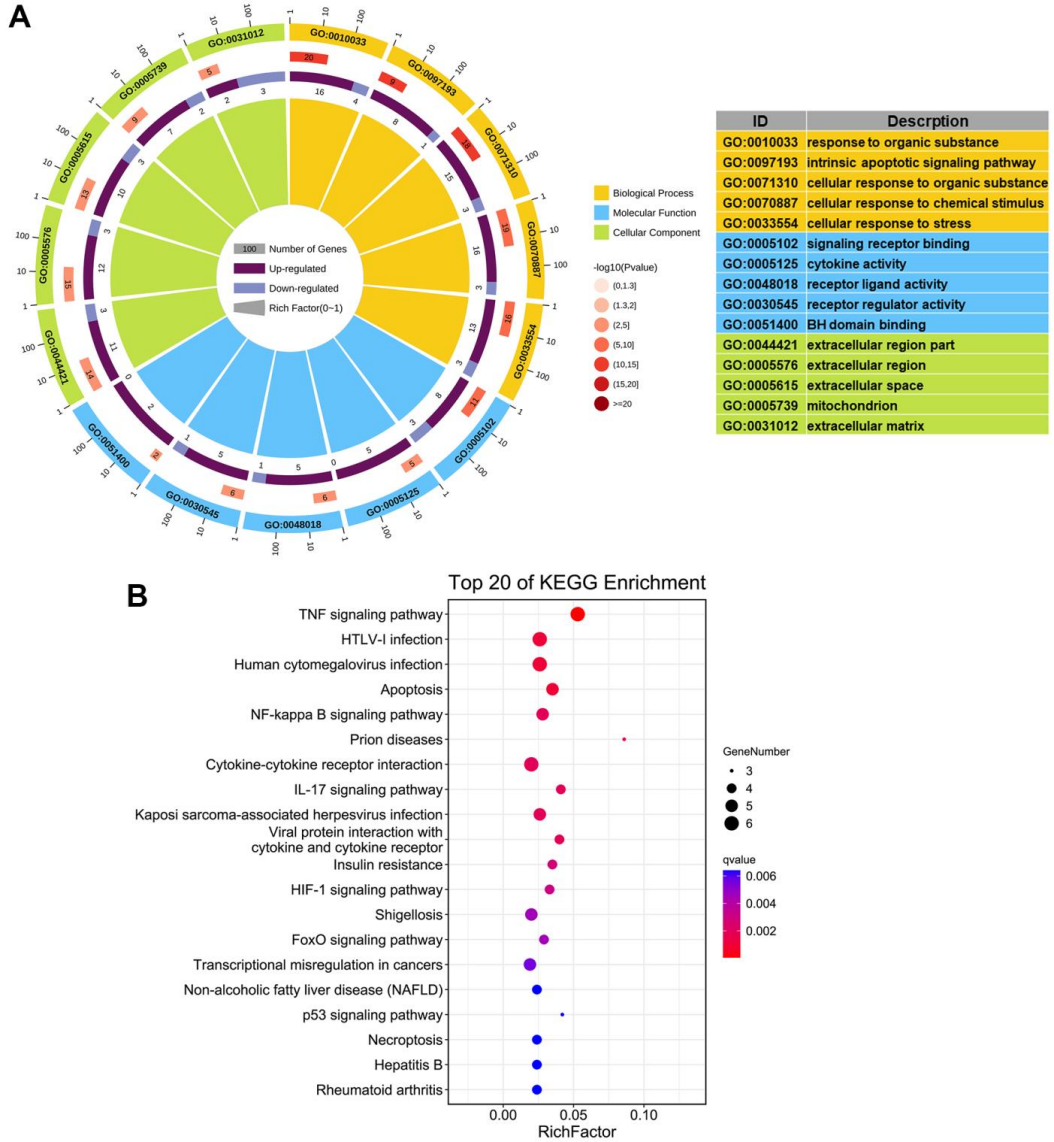
**Functional enrichment analysis of 24 aging- and COPD-related genes.** (**A**) GO enrichment analysis; the top 5 significantly enriched biological processes (*P* < 0.05), molecular functions, and cellular components are listed. (**B**) KEGG analysis; the top 20 significant signaling pathways (*P* < 0.05) are listed.

To explore the potential biological functions of these aging-related genes in COPD, gene ontology (GO) and Kyoto Encyclopedia of Genes and Genomes (KEGG) enrichment analyses were performed (Supplementary Tables 1, 2). Five most significantly enriched GO terms are shown in Figure 4A. The enriched biological processes were cellular response to organic substance, chemical stimulus, stress, and intrinsic apoptotic signaling pathway. The enriched molecular functions were signaling receptor binding, cytokine activity, receptor ligand activity, receptor regulator activity, and BH domain binding. And the enriched cellular components were mitochondrion, extracellular region, extracellular matrix, and extracellular space. KEGG enrichment analysis identified apoptosis, cytokine-cytokine receptor interaction, human cytomegalovirus infection, and insulin resistance as over-represented processes, and TNF, NF-κB, IL-17, and HIF-1 as mainly representative signaling pathways (Figure 4B).

### Validation of the candidate aging-related genes in COPD

**Figure 5:**
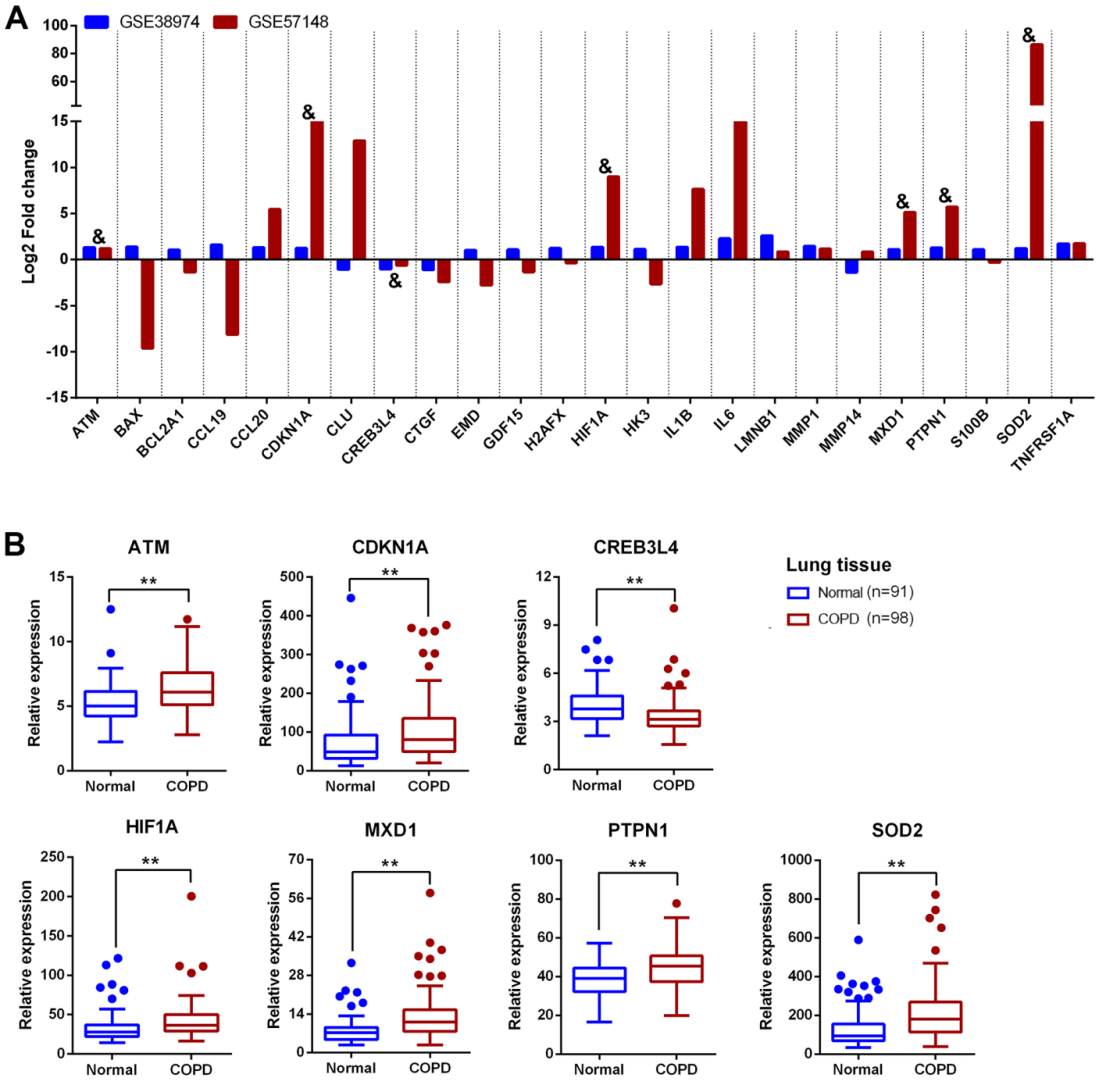
**Validation of 24 aging- and COPD-related genes in GSE57148.** (**A**) The comparative analysis of 24 aging- and COPD-related genes in GSE38974 and GSE57148 datasets; “&” means the genes with consistent relative expression trend in both GSE38974 and GSE57148 datasets. (**B**) Boxplot of the expression of 7 aging- and COPD-related genes (*ATM, CDKN1A, CREB3L4, HIF1A, MXD1, PTPN1*, and *SOD2*) in GSE57148. Data presented as probe intensity. *P <0.05; **P <0.01.

**Figure 6:**
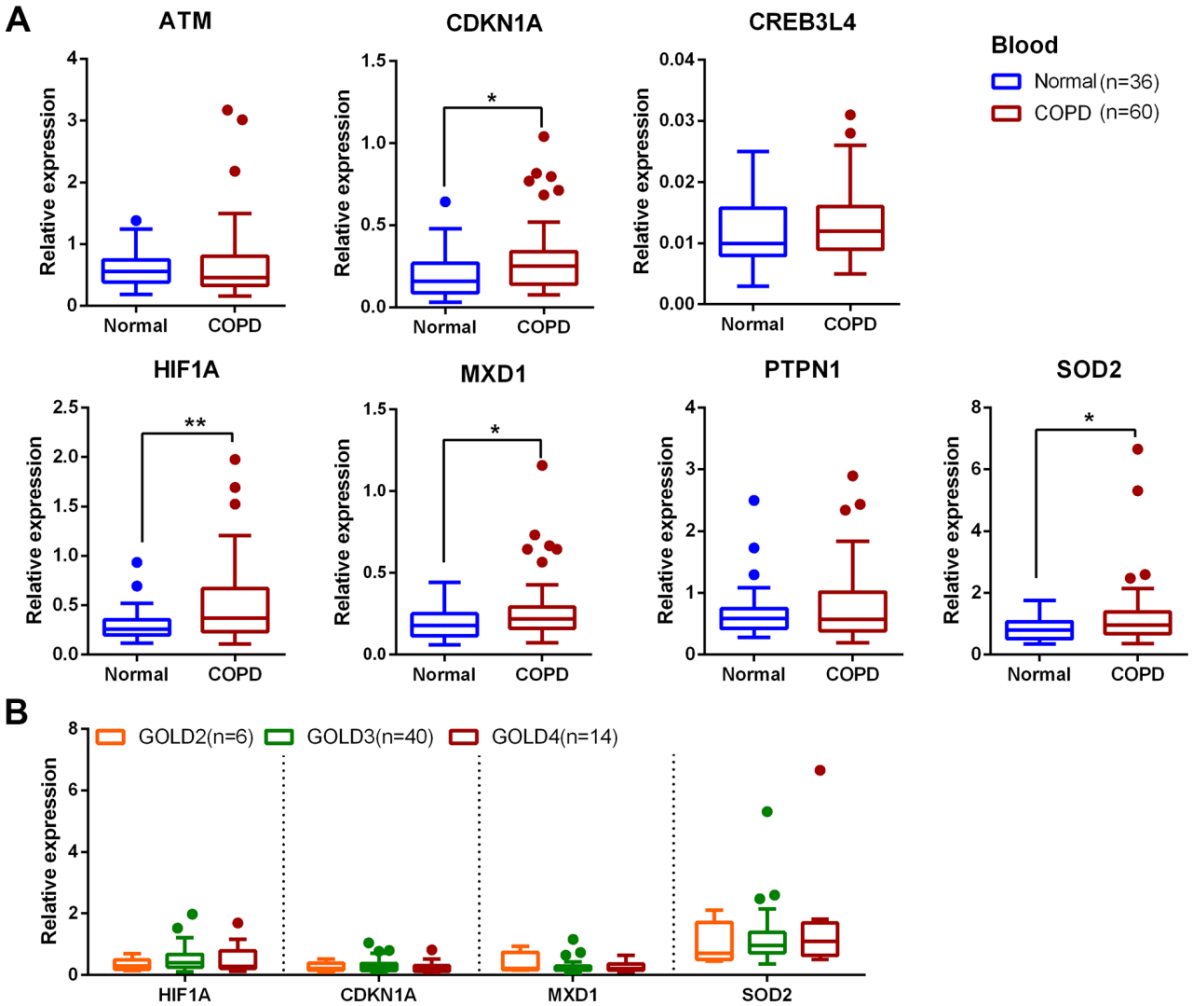
**Expression of 7 aging-related gene candidates in COPD and normal PBMCs.** (**A**) Expression analysis of *ATM, CDKN1A, CREB3L4, HIF1A, MXD1, PTPN1* and *SOD2* in blood samples by qRT-PCR; data are present as 2^(-ΔCт)^ relative to *GAPDH*. (**B**) Expression analysis of *HIF1A*, *CDKN1A, MXD1* and *SOD2* at different GOLD stages of COPD. *P <0.05; **P <0.01.

To validate the 24 candidate genes, their expression profiles were checked in another COPD transcriptomics dataset (GSE57148) containing 91 normal and 98 COPD lung tissue samples. Expression changes of the 24 genes in GSE38974 and GSE57148 are listed in Table 1 and Figure 5. Expression of *ATM, CDKN1A, CREB3L4, HIF1A, MXD1, PTPN1*, and *SOD2* were consistently altered in both datasets. In order to investigate the expression of above genes in blood of COPD patients, we further enrolled 60 COPD patients and 36 normal controls and collecting peripheral blood for validation. The demographic and clinical characteristics of the subjects are summarized in Table 2. The qRT-PCR analysis was confirmed that *CDKN1A, HIF1A, MXD1*, and *SOD2* were significantly upregulated in peripheral blood mononuclear cells (PBMCs) of COPD patients compared with normal controls (Figure 6).

**Table 1:** The analysis of 24 aging- and COPD-related genes in GSE38974 and GSE57148 datasets.

**Gene**	**GSE38974**			**GSE57148**		
	**Log_2_FC**	**P value**	**Type**	**Log_2_FC**	**P value**	**Type**
**ATM**	**1.319**	**0.000**	**Up**	**1.195**	**0.000**	**Up**
BAX	1.388	0.000	Up	-9.594	0.000	Down
BCL2A1	1.059	0.001	Up	-1.350	0.734	Down
CCL19	1.598	0.002	Up	-8.104	0.145	Down
CCL20	1.308	0.040	Up	5.466	0.645	Up
**CDKN1A**	**1.239**	**0.003**	**Up**	**32.441**	**0.010**	**Up**
CLU	-1.059	0.000	Down	12.903	0.235	Up
**CREB3L4**	**-1.023**	**0.000**	**Down**	**-0.620**	**0.001**	**Down**
CTGF	-1.108	0.006	Down	-2.401	0.859	Down
EMD	1.003	0.000	Up	-2.772	0.001	Down
GDF15	1.077	0.013	Up	-1.323	0.765	Down
H2AFX	1.231	0.000	Up	-0.350	0.624	Down
**HIF1A**	**1.356**	**0.000**	**Up**	**8.993**	**0.014**	**Up**
HK3	1.115	0.000	Up	-2.646	0.017	Down
IL1B	1.371	0.032	Up	7.645	0.060	Up
IL6	2.280	0.013	Up	24.521	0.507	Up
LMNB1	2.596	0.000	Up	0.850	0.412	Up
MMP1	1.456	0.038	Up	1.151	0.201	Up
MMP14	-1.358	0.000	Down	0.837	0.725	Up
**MXD1**	**1.092**	**0.001**	**Up**	**5.142**	**0.000**	**Up**
**PTPN1**	**1.294**	**0.000**	**Up**	**5.714**	**0.000**	**Up**
S100B	1.109	0.004	Up	-0.285	0.570	Down
**SOD2**	**1.220**	**0.004**	**Up**	**86.434**	**0.000**	**Up**
TNFRSF1A	1.720	0.000	Up	1.763	0.256	Up

**Table 2:** Clinicopathological characteristics of recruited subjects in this study.

	**Normal(n=36)**	**COPD(n=60)**	***P* Value**
**Gender(female/male)**	15/21	4/56	0.000
**Age(years)**	61.889±7.058	62.883±5.396	0.439
**BMI**	23.638±2.989	21.727±3.360	0.006
**Current/ex-smokers**	12/20	18/51	0.243
**Pulmonary function**			
FEV1 % predicted	101.947±24.852	38.136±10.363	0.000
FEV1/FVC %	81.824±6.598	47.839±10.623	0.000

**Figure 7:**
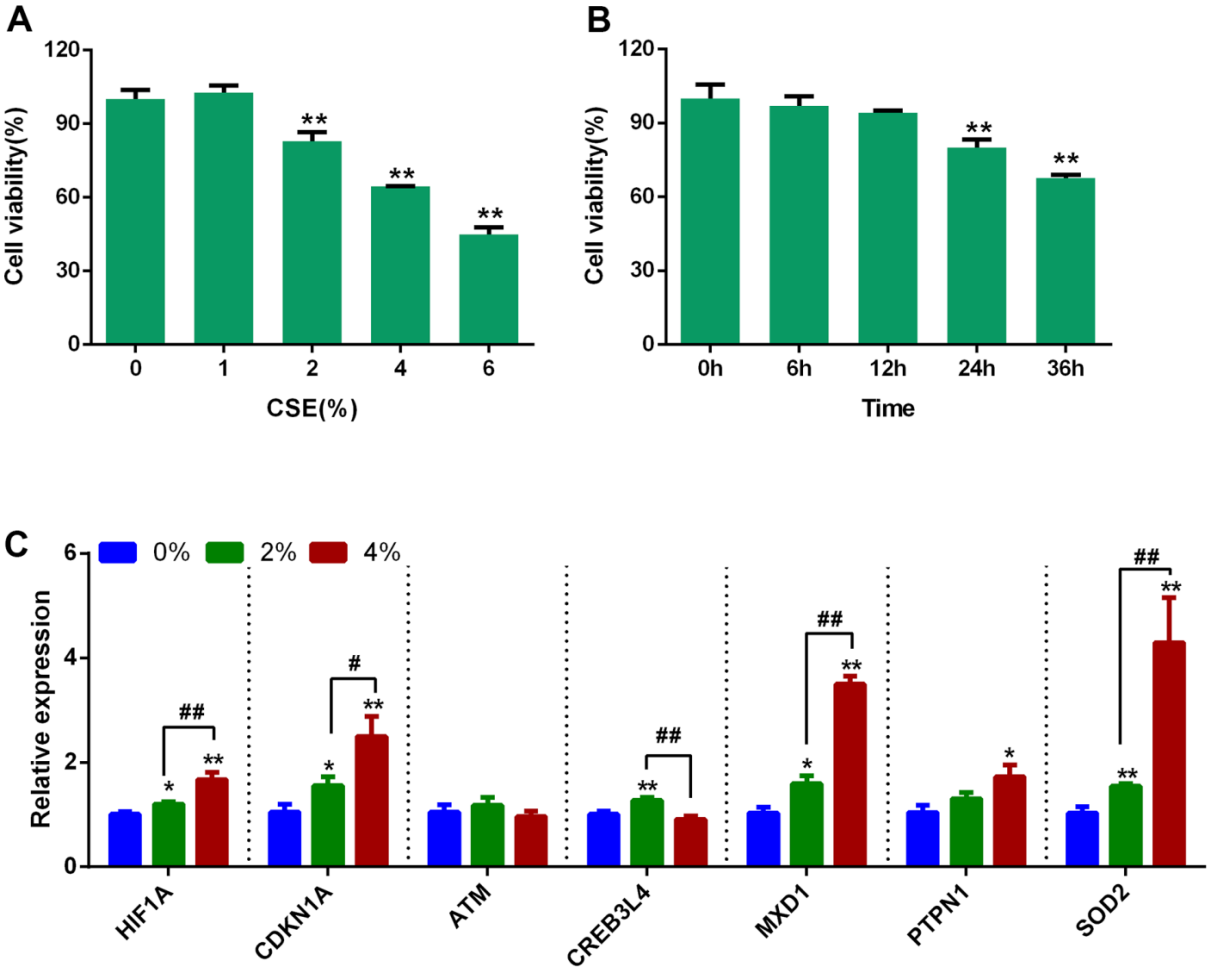
**Expression of 7 aging- and COPD-related genes in CSE-stimulated Beas-2B cells.** (**A**) Effects of different concentrations of CSE on the cell viability of Beas-2B at 24 hours. (**B**) Beas-2B cells were treated with 2% CSE, and the cell viability was measured at different time points. (**C**) Expression analysis of the 7 aging- and COPD-related genes in CSE-stimulated Beas-2B cells; data are presented as 2^(-ΔΔCт)^ relative to *GAPDH*. ^*^P <0.05, **P <0.01 (0% vs. 2% and 4 %); ^#^*P* <0.05, ^##^*P* <0.01 (2% vs. 4%).

The expression levels of *CDKN1A, HIF1A, MXD1*, and *SOD2* were also investigated in Beas-2B cells exposed to cigarette smoke extract (CSE). Firstly, Beas-2B cells were treated with different concentrations of CSE (0, 1, 2, 4 and 6%) and cell viability was measured at 0, 6, 12, 24, and 36 hours. Cell viability was significantly decreased after treatment with 2%, 4%, and 6% CSE for 24 hours, with a dose-dependent effect (Figure 7A, 7B). Therefore, 2% and 4% CSE for 24 hours were used for the qRT-PCR experiments. Expression of *CDKN1A, HIF1A, MXD1*, and *SOD2* were significantly increased after CSE exposure (Figure 7C).

### Diagnostic value and correlation analysis for aging-related genes in COPD

**Figure 8:**
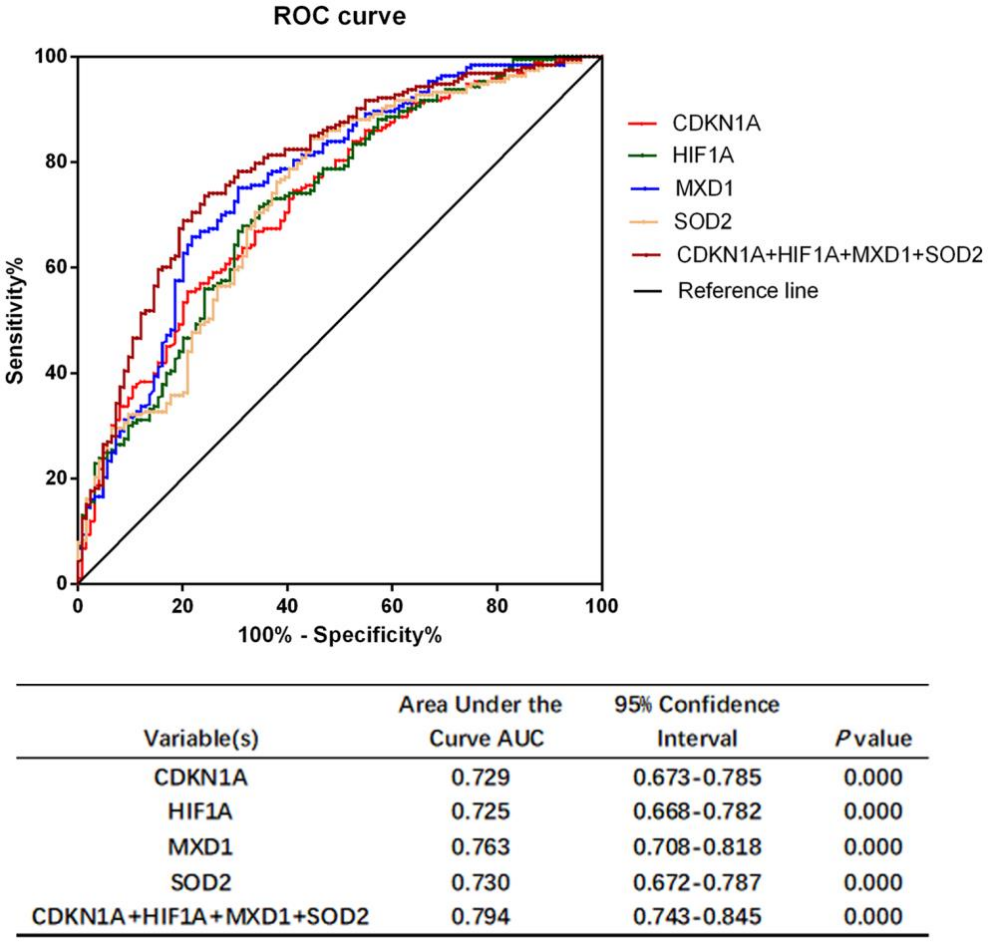
**Performance characteristics of aging- and COPD-related genes in ROC curve analysis.** ROC curve analysis of *CDKN1A, HIF1A, MXD1,* and *SOD2* alone and in combination.

**Figure 9:**
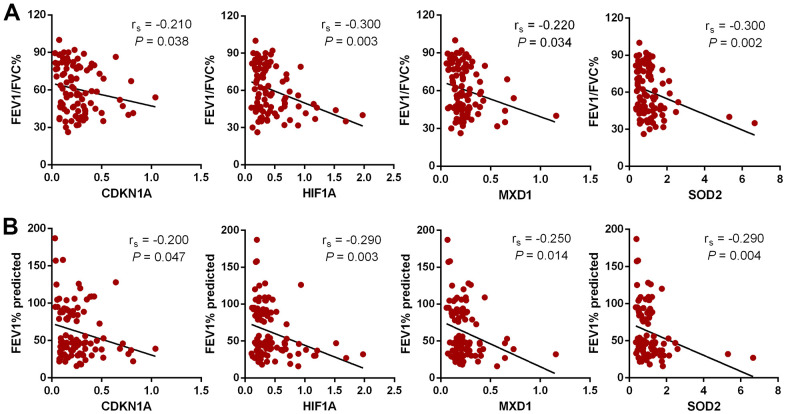
**The relationship between 4 aging- and COPD-related genes and lung function by Spearman rank correlation analysis.** (**A**) Correlation between candidate genes and FEV1/FVC%. (**B**) Correlation between candidate genes and FEV1% predicted.

The ability of the expression of these genes to distinguish COPD subjects from normal controls was investigated by receiver operating characteristic (ROC) curve analysis (Figure 8). The area under the curve (AUC) values for *CDKN1A, HIF1A, MXD1,* and *SOD2* were 0.729 (95% CI 0.673–0.785), 0.725 (95% CI 0.668–0.782), 0.763 (95% CI 0.708–0.818) and 0.730 (95% CI 0.672–0.787), respectively. Combining the expression levels of four genes gave an AUC of 0.794 (95% CI 0.743-0.845), suggesting that the diagnostic value of the combination is slightly better than that of the four individual genes for distinguishing COPD patients from normal subjects. We also investigated the correlation between the expression of each candidate gene and pulmonary function. Expression levels of *CDKN1A, HIF1A, MXD1,* and *SOD2* were negatively correlated with FEV1/FVC% and FEV1% predicted (Figure 9).

### Identification of miRNA upstream of *CDKN1A, HIF1A, MXD1* and *SOD2*

**Figure 10:**
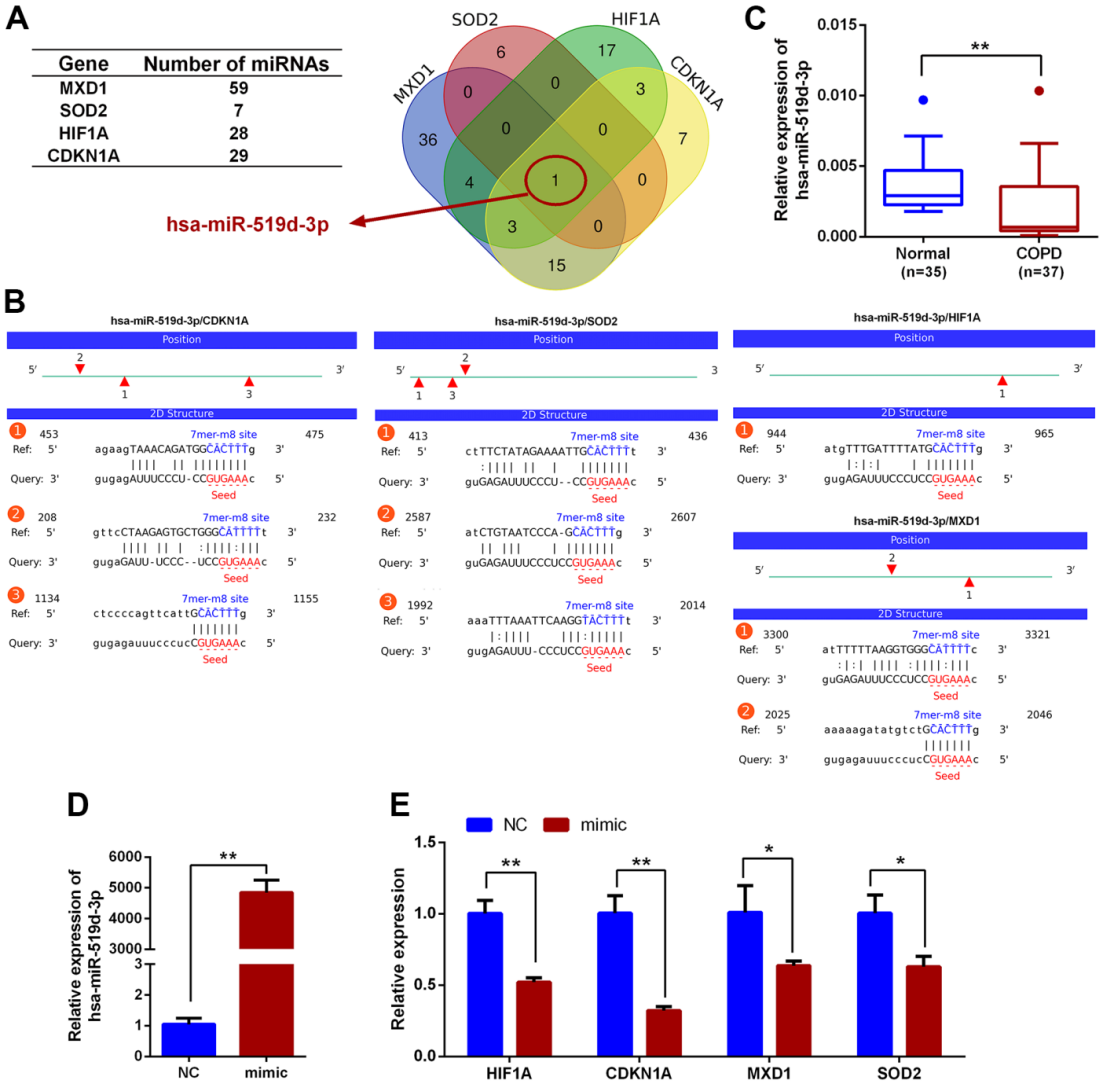
**Prediction of miRNAs that could regulate *HIF1A, CDKN1A, MXD1* and *SOD2*.** (**A**) Venn diagram of miRNAs predicted to target *CDKN1A, HIF1A, MXD1* and *SOD2*. (**B**) The predicted interactions of hsa-miR-519d-3p with *CDKN1A, HIF1A, MXD1* and *SOD2*. (**C**) qRT-PCR expression analysis of hsa-miR-519d-3p in PBMCs from COPD and healthy volunteers; data are presented as 2^(-ΔCт)^ relative to *U6*. (**D**) Expression analysis of hsa-miR-519d-3p in Beas-2B cell following transfection with hsa-miR-519d-3p mimic. (**E**) Expression of *CDKN1A, HIF1A, MXD1* and *SOD2* in Beas-2B cell following transfecting with hsa-miR-519d-3p mimic; data are presented as 2^(-ΔΔCт)^ relative to *GAPDH*. *P <0.05; **P <0.01.

**Figure 11:**
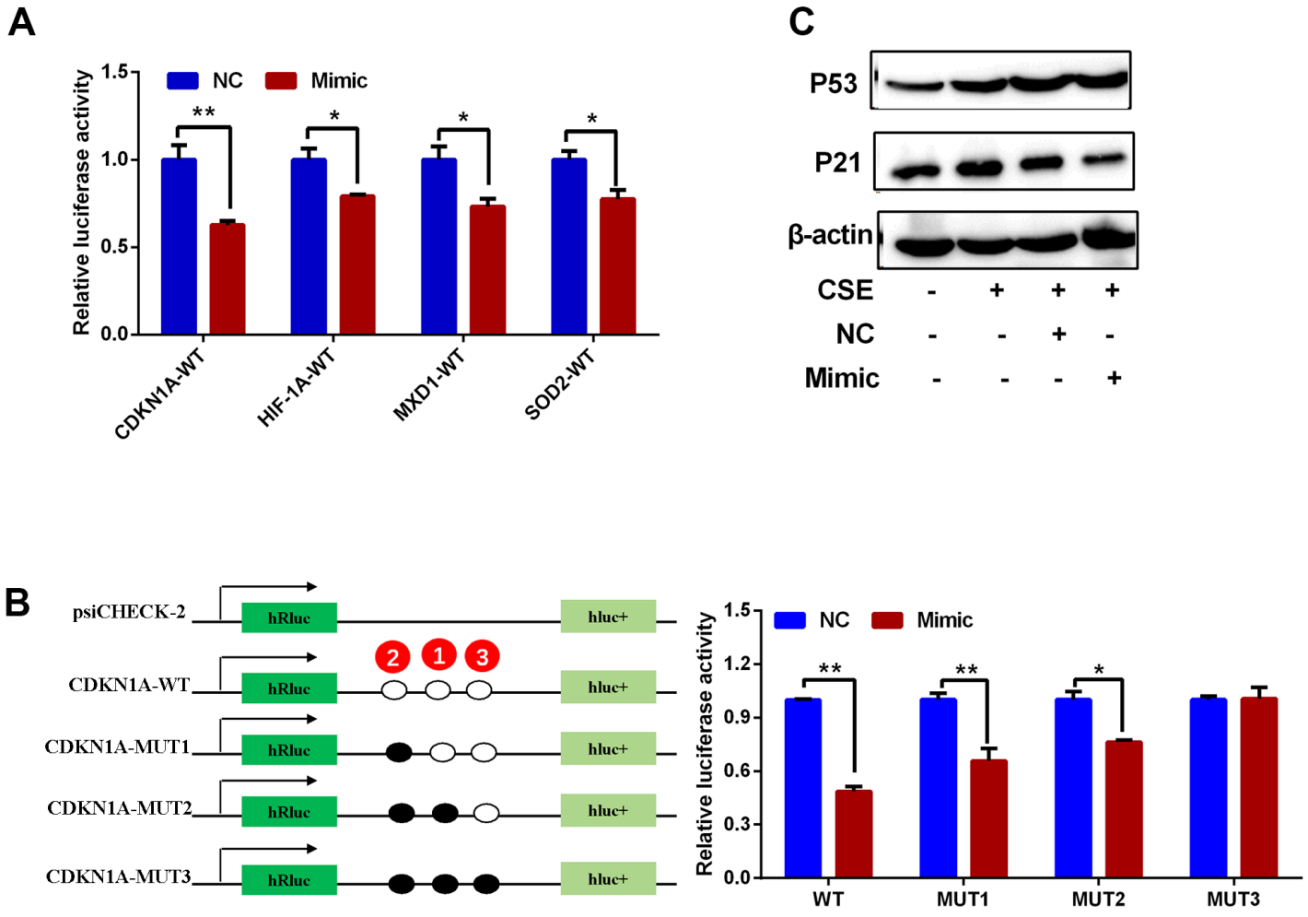
**Regulatory interactions between hsa-miR-519d-3p and *CDKN1A, HIF1A, MXD1* and *SOD2*.** (**A**) Luciferase reporter assay for detection of the interaction of hsa-miR-519d-3p with 4 candidate genes. (**B**) Results of luciferase reporter assay demonstrate that hsa-miR-519d-3p directly binds to *CDKN1A*. (**C**) Western blot analysis of the effect of hsa-miR-519d-3p on CSE-induced aging-related proteins. *P <0.05; **P <0.01.

Upstream miRNAs for *CDKN1A, HIF1A, MXD1*, and *SOD2* were predicted using the StarBase3.0 software, and miRNA candidates that were identified by at least four different target-predicting programs were considered hits. The predicted miRNAs are shown in Supplementary Table 3. There were 29, 28, 59 and 7 miRNAs predicted to target *CDKN1A, HIF1A, MXD1,* and *SOD2*, respectively. hsa-miR-519d-3p was the only miRNA predicted to target all four genes (Figure 10A) with 3, 1, 2, and 3 potential interaction sites on the *CDKN1A, HIF1A, MXD1,* and *SOD2* mRNA sequences, respectively. The seeding matched sequences are shown in Figure 10B. Subsequently, the expression of hsa-miR-519d-3p in PBMCs from COPD and normal groups was quantified by qRT-PCR, with hsa-miR-519d-3p expression significantly decreased in COPD patients, compared with the normal controls (Figure 10C). Furthermore, overexpression of hsa-miR-519d-3p in Beas-2B cell using a mimic (Figure 10D) significantly decreased the expression of *CDKN1A, HIF1A, MXD1,* and *SOD2* (Figure 10E). Luciferase reporter assays were used to investigate the interaction of hsa-miR-519d-3p with the four genes. As shown in Figure 11A, the luciferase activities of psiCHECK2 vectors harboring wild-type 3’UTR sequences for these four genes were all reduced after co-transfection with the hsa-miR-519d-3p mimic, and the *CDKN1A* considerably more reduced than the other three genes. The luciferase activity of psiCHECK2 vectors harboring mutational 3’UTR sequences of *CDKN1A* was also measured, while the luciferase activity not significantly different in the presence of hsa-miR-519d-3p after mutating all predicted interaction sites (Figure 11B). This suggests that hsa-miR-519d-3p can bind to 3’-UTR position of the *CDKN1A* transcript to inhibit expression. Finally, overexpression of has-miR-519d-3p decreased the expression of p21 (the protein encoded by *CDKN1A*) in Beas-2B cells (Figure 11C).

## DISCUSSION

The incidence of COPD is increasing worldwide. Importantly, most elderly patients suffering from COPD show age-related changes of the lung [27–29] accompanied with other chronic diseases such as cardiovascular disease, hypertension, metabolic disorder, cognitive impairment, and gastrointestinal conditions. All of these co-morbidities are linked to aging-associated pathological mechanisms [9, 10, 30, 31]. Although, the underlying mechanisms of COPD are a topic of active research, current therapeutics for COPD are mostly borrowed pharmacological therapy from the treatment of asthma [21], effective therapies are still absent for this irreversible disease, especially for older patients. It is therefore crucial to identify novel therapeutic targets specifically for COPD.

In this study 24 aging- and COPD-correlated genes were identified using a variety of bioinformatic approaches. These 24 genes principally involved in TNF, NF-κB, IL-17, HIF-1 signaling pathways, which were associated with COPD pathophysiology, such as aging, inflammation, and oxidative stress [32–34]. The genes identified in this study (*CDKN1A, HIF1A, MXD1,* and *SOD2*) were significantly upregulated in lung tissues in two different clinical COPD transcriptomics datasets, and had the potential to develop as therapeutics targets of COPD. Although the lung is the principal target organ in COPD, the blood biomarkers are increasingly applied to in diagnosis and prognosis of conditions due to the collection and dynamic monitoring easily. Hence, we validated the above four genes in blood samples from a new third cohort, and have found that the trend of the relative expression of these genes are consistent in blood and lung tissue. Furthermore, the expression of these genes was negatively correlated with pulmonary function in our validated cohorts.

Several studies have already demonstrated that dysregulation of *CDKN1A, HIF1A* and *SOD2* are associated with the aging phenotype in COPD patients [35–41]. For example, *CDKN1A* (which encodes the p21 protein) is an important cyclin-dependent kinase inhibitor, which can potentiate the inflammatory response and cellular senescence, and is reported to be significantly upregulated in COPD [35, 36]. Decreasing the expression of *CDKN1A* can attenuate multiple pro-inflammatory stimuli-mediated lung oxidative and inflammatory responses and plays a critical role in cigarette smoke induced senescence of lung cells during COPD pathogenesis [36]. Zhang et al. identified 40 potential autophagy-related genes which were differentially expressed in COPD, including *CDKN1A* and *HIF1A*, and suggested that overexpression of *CDKN1A* and *HIF1A* might be implicated in the pathogenesis of COPD by regulating autophagy [37]. In addition, *HIF1A* expression was increased in the airway epithelial cells of COPD patients and serves as an important transcriptional regulator to promote the cellular response to inflammatory and oxidative stress [38, 39]. *SOD2*, an antioxidant-related gene, is found significantly increased in alveolar macrophages after exposure to fine atmospheric particulate matter [40]. Another study indicates that the expression of SOD2 protein is increased in bronchial epithelial cells from COPD donors following infection with rhinovirus [41]. *MXD1* is a transcription factor that belongs to the MYC/MXD/MAX family, and is implicated in the pathophysiology of avian influenza virus infections [42], intracerebral hemorrhage [43], and various cancers such as osteosarcoma, lung adenocarcinoma and B cell lymphoma [44–46]. *MXD1* can antagonize the transcriptional activation of c-Myc, serving as a transcription repressor [47, 48]. It can also be regulated by miRNAs to form a potential tumor-suppressing positive feedback loop [49]. However, *MXD1* has not been studied in COPD. Future functional research is required to investigate the contribution of *MXD1* to COPD development.

The severity of COPD is largely related to symptoms, exacerbations and comorbidities, while underdiagnosis of COPD is a common problem worldwide. The current diagnosis of COPD is depending on spirometry and patients’ demographic characteristics that could contributed to overdiagnosis or misdiagnosis aperiodically [50]. Diagnostic tests based on biomarker combinations are often evaluated by the area under the ROC curve analysis to discriminate individuals who have disease and individuals who are disease-free to assisting the clinical diagnosis [51]. In this study, the AUC values of *CDKN1A, HIF1A, MXD1,* and *SOD2* were all greater than 0.7, and the combination of the four genes showed a better discrimination (AUC = 0.794). This suggested that *CDKN1A, HIF1A, MXD1,* and *SOD2* had excellent discriminate ability between COPD patients and health subjects. However, whether these candidate genes can be applied to clinical practice for prevention of and intervention into COPD development is currently unclear, further studies are required to explore the molecular mechanisms of COPD *in vivo* and *in vitro*.

Previous studies have identified miRNA-based therapeutics, applied to clinical testing, and dysregulated miRNAs in COPD, such as miR-195 and miR-181c, which have potential promise in alleviating COPD *in vivo*, and may serve as therapeutic targets for COPD in the near future [18, 20, 21]. Therefore, we identified miRNAs potentially upstream of *CDKN1A, HIF1A, MXD1,* and *SOD2* expression changes. hsa-miR-519d-3p was predicted to target *CDKN1A, HIF1A, MXD1*, and *SOD2*, and its expression was significantly downregulated in COPD. Previous studies have reported that hsa-miR-519d-3p is involved in tumorigenesis in gastric cancer, pancreatic cancer, and lung cancer [52–54], and correlates with cell proliferation and migration in trophoblastic cell lines [55]. The biological function of hsa-miR-519d-3p in COPD is unknown. In this study, hsa-miR-519d-3p was shown to directly bind to *CDKN1A*, expression of which was associated with the aging phenotype in COPD patients. Further in-depth study of hsa-miR-519d-3p may provide a new biomarker for COPD.

In conclusion, this study identified hsa-miR-519d-3p and four aging- and COPD-related genes (*HIF1A, CDKN1A, MXD1*, and *SOD2*) that may influence the development of COPD. Further study of these new targets may lead to new insight into the pathogenesis, diagnosis, and treatment of COPD.

## MATERIALS AND METHODS

### Aging-related genes and mRNA expression in COPD

A total of 500 aging-related genes were obtained from the Aging Atlas database (https://ngdc.cncb.ac.cn/aging/index). Lung gene expression data from a study of 9 normal and 23 COPD patients (GSE38974), and a study of 91 normal and 98 COPD patients (GSE57148) were obtained from the GEO database (http://www.ncbi.nlm.nih.gov/geo/).

### Selection of aging- and COPD-correlated genes

Differentially expressed genes were identified in the GSE38974 dataset using the DESeq2 R package. Adjusted *P* < 0.05 and |log_2_ fold change (FC)| > 1 were used as a threshold for significantly differential expression. Aging-related genes in this set of COPD differentially expressed genes were identified using the 500 genes from the Aging Atlas database.

### Biological function and signaling pathway enrichment analysis

A webserver (https://www.omicshare.com/) was used to functionally annotate the genes that were associated with aging and COPD. GO enrichment analysis was used to identify enriched molecular functions, cellular components, and biological processes. KEGG enrichment analysis was used to identify signaling pathways enriched in the list of aging- and COPD-related genes.

### Enrolment of subjects and collection of samples

A total of 60 COPD patients and 36 aging-matched healthy controls were enrolled from the first affiliated hospital of Wenzhou medical university from December 2017 to December 2019. All participants were asked to read a document approved by the Human Medical Ethics Committee of the First Affiliated Hospital of Wenzhou Medical University (approval no.: 2016131) and written informed consent was provided by all participants. All participants were aged 40–80 years old and COPD patients suffered from respiratory symptoms, and fitted the GOLD diagnostic criteria. Patients with other complications such as cancer, cardiac conditions, and other respiratory diseases (e.g., bronchiectasis, bronchial asthma pulmonary fibrosis and/or active tuberculosis) were excluded. Peripheral blood samples were collected, and peripheral blood mononuclear cells (PBMCs) were isolated from 10 mL blood samples using human lymphocyte separation medium (Solarbio, China). All PBMCs samples were immediately stored at -80° C.

### Preparation of cigarette smoke extract (CSE) and cell cultures

Two unfiltered cigarettes (0.8 mg of nicotine and 9 mg of tar per cigarette) were used to bubble smoke into 10 mL Dulbecco’s modified Eagle’s medium (DMEM; Gibco, USA) at a speed of 2 minutes per cigarette. The medium was sterilized using a 0.22 μm filter. This solution was considered as a 100% CSE solution to apply in subsequent experiments.

Human bronchial epithelial cells (Beas-2B) were cultured in DMEM supplemented with 10% fetal bovine serum (FBS; Gibco, USA) and under a humidified atmosphere of 5% CO2 at 37° C. The cells were grown to approximately 90% confluence before experiments using CSE or transfection with 40 nM hsa-miR-519d-3p mimic or negative control (NC) oligoribonucleotides (Sangon Biotech, China) using Lipofectamine™ 3000 (Invitrogen, USA) according to the manufacturer’s instructions.

### Cell viability assay

Beas-2B cells were seeded into 96-well plates at a density of 5 × 10^3^ cells/well and incubated overnight, then treated with CSE at 0, 1, 2, 4 and 6%. Cell viability was measured at different times (0, 6, 12, 24, and 36 hours) using cell counting kit-8 (CCK-8) reagent (YEASEN, China) according to the manufacturer’s instructions. After an incubation with CCK-8 reagent for 2 hours, the optical density was measured at 450 nm using a microplate reader (Bio-Rad Laboratories, USA). Cell viability was calculated by the following formula: (absorbance of treatment group/absorbance of control group) × 100%.

### Quantitative real-time PCR (qRT-PCR)

Total RNA was extracted from PBMCs or cultured cells using M5 Hiper Universal Plus RNA Mini Kit (Mei5 Biotechnology, China). cDNA was synthesized using the cDNA synthesis kit or Mir-X miRNA First-Strand Synthesis Kit (both TaKaRa, Japan). Primers for qRT-PCR were designed via a public resource named PrimerBank (https://pga.mgh.harvard.edu/primerbank/) and synthesized by Sangon Biotech, which are listed in Table 3. qRT-PCR amplification was performed using SYBR Green PCR Premix Ex Taq™ II reagents (TaKaRa, Japan) with the Quant Studio 6 FlexI real-time PCR system (Applied Biosystems, USA), following the protocols from the commercial kits. Expression levels of tested genes were determined with the 2^-ΔCt^ or 2^-ΔΔCt^ method based on the endogenous control (*GAPDH* or U6).

**Table 3:** Primer sequences for validating gene candidates.

**Gene name**	**Primer sequence (5’-3’)**	
	**Forward primer**	**Reverse primer**
HIF1A	ATCCATGTGACCATGAGGAAATG	TCGGCTAGTTAGGGTACACTTC
CDKN1A	TGTCCGTCAGAACCCATGC	AAAGTCGAAGTTCCATCGCTC
ATM	GGCTATTCAGTGTGCGAGACA	TGGCTCCTTTCGGATGATGGA
CREB3L4	CAGACGCTAATTGCTCAAACTTC	CCACTTGGGTCTCCAGATTTTCT
MXD1	CGTGGAGAGCACGGACTATC	CCAAGACACGCCTTGTGACT
PTPN1	TCCCTTTGACCATAGTCGGAT	GTGACCGCATGTGTTAGGCA
SOD2	TTTCAATAAGGAACGGGGACAC	GTGCTCCCACACATCAATCC
GAPDH	CAATGACCCCTTCATTGACC	TTGATTTTGGAGGGATCTCG
hsa-miR-519d-3p	CAAAGTGCCTCCCTTTAGAGTG	

### Prediction of upstream miRNAs

StarBase3.0 (http://starbase.sysu.edu.cn/), which includes seven miRNA target prediction programs (PITA, RNA22, miRmap, DIANA-microT, miRanda, PicTar and TargetScan) was used to predict the miRNAs that may regulate the genes we identified. Hits were chosen which appeared in at least 4 of the 7 prediction programs. The interactions between miRNA and mRNA were visualized using Miranda software.

### Luciferase activity assay

The psiCHECK2 luciferase reporter vectors (Promega, USA) harboring wild-type (WT) or mutated (MUT) 3’UTR sequences were co-transfected with hsa-miR-519d-3p mimic or the corresponding negative control (NC) into human embryonic kidney (HEK) 293T cells. After a 48-hour incubation, cells were lysed and luciferase activity was measured using a dual-luciferase reporter assay system (Promega, USA), according to the manufacturer’s protocol.

### Western blot

Total proteins were extracted from Beas-2B cells by using radio-immunoprecipitation assay (RIPA) buffer with protease inhibitor (Roche Applied Science, USA), then the protein concentration was measured using a BCA kit (Thermo, USA). Protein samples were separated on 12% SDS-PAGE gels at 80 V for 120 minutes and transferred onto a nitrocellulose membrane (Millipore Co, USA) at 300 mA for 1 hour using a wet transfer method. The membranes were incubated with primary antibody against p21 (10355-1-AP; Proteintech, USA), p53 (#2524; Cell Signaling Technology, USA) and β-actin (#3700; Cell Signaling Technology, USA) at 4° C overnight. Horseradish peroxidase (HRP)-conjugated secondary antibodies (A0208 and A0216; Beyotime, China) were incubated at room temperature for 1 hour, and visualized using an enhanced chemiluminescence kit and Image Lab (Bio-Rad, USA).

### Statistical analysis

GraphPad Prism 6.0 (GraphPad Software Inc., San Diego, CA, USA) and SPSS 21.0 were used for statistical analysis. Student's *t*-test or Mann-Whitney U-test was applied for analyzing the data between two groups based on the normality of data. Spearman’s rank correlation coefficient was used to investigate the correlation between gene expression and pulmonary function of COPD patients. The area under the curve (AUC) of the receiver operating characteristics curve (ROC) was evaluated to assess effectiveness in discriminating patients with COPD from healthy participants. *P* <0.05 was considered statistically significant.

### Availability of data and material

The data used to support the findings of this study are available from the corresponding author upon request.

## Supplementary Material

Supplementary Table 1

Supplementary Table 2

Supplementary Table 3
