# Supplementary material for: Identification and validation of aging-related genes in COPD based on bioinformatics analysis
Source: Aging (Albany NY). 2022 May 24;14(10):4336–56. doi: 10.18632/aging.204064 (PMC9186770; doi:10.18632/aging.204064)
Supplement: Supplementary Table 2 [file aging-14-204064-s002.pdf]

SUPPLEMENTARY TABLE

Supplementary Table 2. KEGG analysis.

ID	Description	Class	Up	Down	num	qvalue
ko04668	TNF signaling pathway	Environmental Information Processing	4	2	6	0.000054
ko05166	HTLV-I infection	Human Diseases	5	1	6	0.001050
ko05163	Human cytomegalovirus infection	Human Diseases	5	1	6	0.001050
ko04210	Apoptosis	Cellular Processes	5	0	5	0.001050
ko04064	NF-kappa B signaling pathway	Environmental Information Processing	5	0	5	0.001880
ko05020	Prion diseases	Human Diseases	3	0	3	0.001880
ko04060	Cytokine-cytokine receptor interaction	Environmental Information Processing	6	0	6	0.001880
ko04657	IL-17 signaling pathway	Organismal Systems	4	0	4	0.001880
ko05167	Kaposi sarcoma-associated herpesvirus infection	Human Diseases	5	0	5	0.001880
ko04061	Viral protein interaction with cytokine and cytokine receptor	Environmental Information Processing	4	0	4	0.001880
ko04931	Insulin resistance	Human Diseases	3	1	4	0.002730
ko04066	HIF-1 signaling pathway	Environmental Information Processing	4	0	4	0.003150
ko05131	Shigellosis	Human Diseases	5	0	5	0.004580
ko04068	FoxO signaling pathway	Environmental Information Processing	4	0	4	0.004580
ko05202	Transcriptional misregulation in cancers	Human Diseases	5	0	5	0.005560
ko04932	Non-alcoholic fatty liver disease (NAFLD)	Human Diseases	4	0	4	0.006410
ko04115	p53 signaling pathway	Cellular Processes	3	0	3	0.006410
ko04217	Necroptosis	Cellular Processes	4	0	4	0.006410
ko05161	Hepatitis B	Human Diseases	3	1	4	0.006410
ko05323	Rheumatoid arthritis	Human Diseases	4	0	4	0.006410
ko01524	Platinum drug resistance	Human Diseases	3	0	3	0.006770
ko05164	Influenza A	Human Diseases	4	0	4	0.007550
ko05165	Human papillomavirus infection	Human Diseases	4	1	5	0.011500
ko04211	Longevity regulating pathway - mammal	Organismal Systems	2	1	3	0.011700
ko04933	AGE-RAGE signaling pathway in diabetic complications	Human Diseases	3	0	3	0.014200
ko05132	Salmonella infection	Human Diseases	4	0	4	0.014600
ko04928	Parathyroid hormone synthesis, secretion and action	Organismal Systems	1	2	3	0.014900
ko05142	Chagas disease (American trypanosomiasis)	Human Diseases	3	0	3	0.014900
ko01523	Antifolate resistance	Human Diseases	2	0	2	0.015100
ko04659	Th17 cell differentiation	Organismal Systems	3	0	3	0.015400
ko04215	Apoptosis - multiple species	Cellular Processes	2	0	2	0.016000
ko05332	Graft-versus-host disease	Human Diseases	2	0	2	0.018500
ko05152	Tuberculosis	Human Diseases	4	0	4	0.018900
ko05216	Thyroid cancer	Human Diseases	2	0	2	0.019300
ko05219	Bladder cancer	Human Diseases	2	0	2	0.022800
ko05130	Pathogenic Escherichia coli infection	Human Diseases	4	0	4	0.025000
ko05162	Measles	Human Diseases	3	0	3	0.027600
ko05160	Hepatitis C	Human Diseases	3	0	3	0.033800
ko04218	Cellular senescence	Cellular Processes	3	0	3	0.033800
ko05144	Malaria	Human Diseases	2	0	2	0.033800
ko05014	Amyotrophic lateral sclerosis (ALS)	Human Diseases	2	0	2	0.035600
ko05134	Legionellosis	Human Diseases	2	0	2	0.035600
ko05213	Endometrial cancer	Human Diseases	2	0	2	0.040200
ko04623	Cytosolic DNA-sensing pathway	Organismal Systems	2	0	2	0.040200

ko05217	Basal cell carcinoma	Human Diseases	2	0	2	0.040200
ko05200	Pathways in cancer	Human Diseases	5	0	5	0.041500
ko00524	Neomycin, kanamycin and gentamicin biosynthesis	Metabolism	1	0	1	0.041500
ko05223	Non-small cell lung cancer	Human Diseases	2	0	2	0.043800
ko05321	Inflammatory bowel disease (IBD)	Human Diseases	2	0	2	0.043800
ko05230	Central carbon metabolism in cancer	Human Diseases	2	0	2	0.047400
ko05211	Renal cell carcinoma	Human Diseases	2	0	2	0.047400
ko05218	Melanoma	Human Diseases	2	0	2	0.047400
ko05203	Viral carcinogenesis	Human Diseases	2	1	3	0.047400
ko05133	Pertussis	Human Diseases	2	0	2	0.048100
ko05214	Glioma	Human Diseases	2	0	2	0.048100
ko05220	Chronic myeloid leukemia	Human Diseases	2	0	2	0.048100
ko05212	Pancreatic cancer	Human Diseases	2	0	2	0.049600
